# Cohen-mansfield agitation inventory total score as a measure of agitation and aggression in Alzheimer’s disease: A factor analysis

**DOI:** 10.1016/j.inpsyc.2025.100056

**Published:** 2025-03-18

**Authors:** Suzanne B. Hendrix, Mary Sano, Constantine Lyketsos, Paul B. Rosenberg, Anton P. Porsteinsson, Bruce L. Brown, Dawson Hedges, Jeffrey L. Cummings

**Affiliations:** a Pentara Corporation, Salt Lake City, UT, USA; b Icahn School of Medicine at Mount Sinai, New York, NY, USA; c James J. Peters VA Medical Center, Bronx, NY, USA; d Johns Hopkins University School of Medicine, Baltimore, MD, USA; e University of Rochester School of Medicine and Dentistry, Rochester, NY, USA; f Brigham Young University, Provo, UT, USA; g Chambers-Grundy Center for Transformative Neuroscience, Department of Brain Health, School of Integrated Health Sciences, University of Nevada, Las Vegas, NV, USA

**Keywords:** Alzheimer’s disease, Agitation and aggression, Clinical outcome assessment, Cohen-Mansfield Agitation Inventory (CMAI)

## Abstract

**Background:**

Alzheimer’s disease (AD) is often associated with agitation and aggression, which may impair function, impede care, and be a major source of stress for caregivers. The Cohen-Mansfield Agitation Inventory (CMAI) is often used to assess agitation and aggression. In its original, nursing-home version, it is a 29-item, caregiver-informed, clinician-administered 7-point scale that assesses the frequency of various agitation or aggressive behaviors. However, the instruction manual advises against the use of the total score in favor of a domain-based analysis. This recommendation has been followed in both clinical trials and practice. Because the CMAI is comprehensive and easy to administer, we sought to determine the validity of its total score as a single construct for assessing agitation and aggression in patients with AD.

**Methods:**

We used a previously conducted factor analysis of the CMAI scores from two risperidone trials in patients with dementia (N = 648), and a follow-up analysis of the subset of patients with psychosis of AD (N = 479), to examine, using vector analysis and an effect-size-versus-signal-to-noise ratio analysis, whether the total CMAI score could confidently be used as a global measure of agitation and aggression in AD.

**Results:**

Our findings suggest that the CMAI items from the dataset analyzed load into 4 clusters, which cover about 50 % of the total data variance. Surprisingly, items with the lowest signal-to-noise ratio (hitting, performing repetitious mannerisms, aimless pacing or wandering) had the strongest response to treatment (and vice versa), and belonged to different factors. The further observation that many items were spread among the factors, instead of primarily measuring a single factor or domain, suggests that there is a continuum of symptoms, and separating them into domains requires separating very similar items that measure two or more domains.

**Conclusions:**

These findings suggest that assessing agitation and aggression via CMAI domains instead of the total score is likely to miss important behavioral signals. Using total CMAI score in clinical trials and practice, along with the assessment of individual items, is warranted.

## Introduction

Agitation or aggression affect up to 60 % of patients with Alzheimer’s disease (AD), with the prevalence increasing with the disease severity [29,30]. These symptoms, which typically present as distress and heightened motor activity plus verbal or physical aggression [8], can impair function, increase caregiver stress, and significantly augment the burden and costs of care. Agitation could be underdiagnosed in outpatient settings [13], as its assessment typically relies on caregivers, whose recall or understanding of the concept of agitation might vary considerably.

Despite a substantial impact of agitation and aggression on patients with AD and their caregivers, there is no standard way of assessing those symptoms in clinical trials or practice. Several measures have been used in clinical trials, including the Cohen-Mansfield Agitation Inventory (CMAI). The CMAI is a validated, clinician-administered, caregiver-informed scale designed to measure agitated and aggressive behavior in patients with AD residing in nursing homes (29 items) or the community (31 items) [7]. (An abbreviated, 14-item version is also in use.) Score range for the nursing-home version is 29–203, with higher scores indicating worse behavioral disturbance. However, after decades of availability, the CMAI total score is still not widely used in research or clinical practice. The CMAI instruction manual states that, “for analysis purposes, it is not useful to calculate a total score by adding all the categories” and recommends aggregating the items in domains that are appropriate for the sample studied [6]. These agitation domains are very similar to those recommended by a recent consensus panel [28].

Assessing individual aspects of agitated or aggressive behavior may be appealing from the individual patient’s perspective, but from the standpoint of clinical research, having a single, holistic measure of these behaviors may be preferable. For example, a single score based on all the CMAI items could improve the instrument’s ability to capture changes in agitation and aggression in a pool of patients in whom manifestations of these behaviors may differ. Here, it should be pointed out that agitation and aggression are typically assessed within the same instrument (eg, CMAI) or within the same item of an instrument (eg, Neuropsychiatric Inventory [NPI]) [31], and that the co-occurrence of those two constructs in patients with dementia can exceed 60 % [5]. A total score might also have advantages as a clinical trial outcome measure, especially in studies that are smaller or focus on patients with early AD. In addition, grouping of individual items into domains may not be equally valid for each patient, and splitting a single score into subscores is associated with a loss of statistical power. Available factor analyses of CMAI data in patients with AD have identified three or four factors, with about half of variance remaining unexplained [16,23]. In addition, various domain scores might not assess agitation and aggression on their own but only in concert. Although several studies have used the CMAI total score as an endpoint [1], controversy remains as to the best method of summarizing the CMAI items.

The purpose of this post hoc analysis was to evaluate the statistical validity of the total CMAI score as a measure of agitation and aggression in individuals with psychosis of AD.

## Methods

### Data

We used the factor analysis of CMAI items from a previous post hoc evaluation [23], in which the authors combined data from three 12- or 13-week randomized, double-blind, placebo-controlled trials of risperidone in patients with psychosis and dementia who were residing in nursing homes (Study 1: Australia and New Zealand, n = 345 [2]; Study 2: Europe and Canada, n = 344 [9]; Study 3: United States, n = 625 [19]) (Table 1). In all 3 studies, patients had at least a minimal level of behavioral and psychological symptoms associated with dementia.

The approach used by Rabinowitz et al. was twofold. In the first, discovery phase, they subjected CMAI data from Study 1 [2] and Study 2 [9] to a principal component analysis, which maximizes variance, with an oblique Promax rotation. An oblique rotation allows principal components (i.e., factors) to be correlated, which is likely closer to reality than the orthogonal rotation, which assumes absence of correlation [12]. The resulting 4-factor structure is summarized in Table 2, showing the loading for each item as a measure of the strength of the relationship with each factor and the variance explained by each factor. In the second, validation phase, they confirmed the existence of identified factors using Study 3 data [19].

In our analysis, we used baseline and endpoint data for each CMAI item from placebo- and risperidone-treated patients in Studies 1 and 2, obtained from the original study publications [2,9].

In addition, we used a different analysis of the same 3 studies, which focused on the subset of patients who met the criteria for psychosis of AD and the 25 CMAI physical items that had a clinically meaningful level at baseline and had at least 45 patients per treatment group [24]. This analysis was chosen because it included changes from baseline for each physical CMAI item, which allowed for the assessment of the relationship between signal-to-noise ratio for those items versus effect size (see below).

### Statistical analysis

Our analysis approach was twofold. First, we created 3-dimensional vector plots of factors [3] obtained with a principal-components extraction (PCA) with Promax rotation in SPSS 12.0 in the discovery stage of the Rabinowitz et al. analysis to visualize the contribution of each CMAI item and their factors to the overall CMAI construct (i.e., the total CMAI score). Of note, we used orthogonal axes although an oblique rotated factor pattern was presented (since the factors were mostly independent) and omitted the plotting of the vectors corresponding to the items within factor 4, because there were only two (hiding things and hoarding things).

Second, using the data for patients with psychosis of AD, we calculated for each included CMAI item (including hiding things and hoarding things) a signal-to-noise ratio of baseline-to-endpoint score change among the placebo-treated patients, as well as a baseline-to-endpoint effect size of risperidone versus placebo and examined the correlation between the two. Specifically, the mean to standard deviation ratio (MSDR) was used as the measure of signal-to-noise ratio. The MSDRs for each item in the CMAI was calculated as the mean change from baseline on each item divided by the corresponding standard deviation in change from baseline [15]. The MSDR is the inverse value of the coefficient of variation. Second, the treatment effect size used a Cohen’s d, was calculated as a difference between risperidone and placebo in mean change from baseline to endpoint divided by the pooled standard deviation. With this approach, an increase (i.e., worsening) of the mean item score in the risperidone group from baseline to endpoint that was one-fourth (0.25) of the pooled standard deviation would correspond to Cohen’s d effect size of 0.25. A change of zero in the CMAI item score in the risperidone group (with any pooled standard deviation) would be equivalent to a Cohen’s d effect size of 0.

## Results

**Fig. 1.**
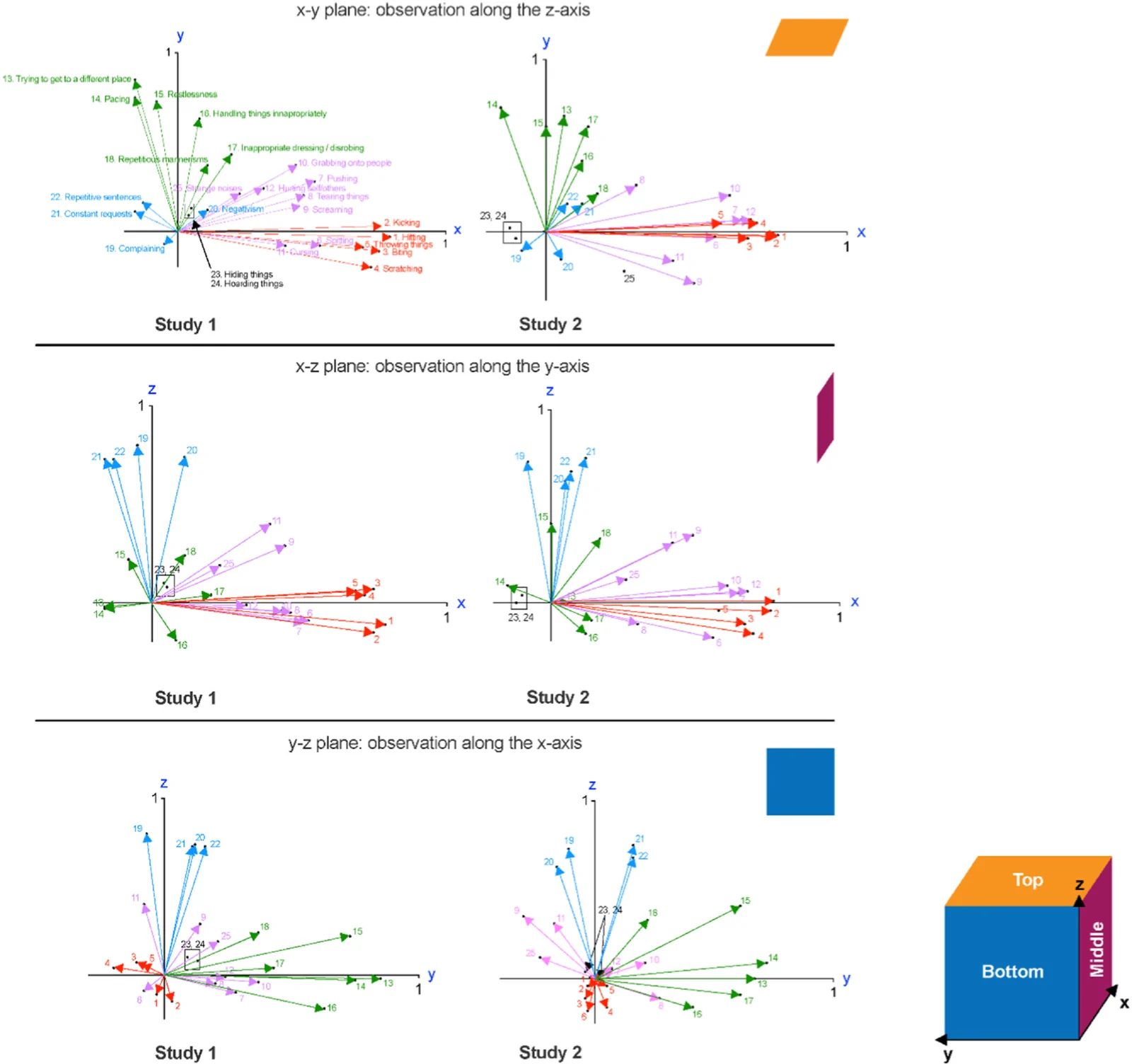
Three-dimensional vector plots of CMAI items in Study 1 and Study 2, as analyzed by Rabinowitz et al. [23]. Items in the clusters of Aggression (red), Restlessness (green), and Negativity (blue) predominant align with the x-, y-, and z-axes of the vector space, respectively. Items from the Combination cluster (pink) are visible to a similar extent from each of the 3 perspectives.

Geometrical similarity of CMAI factor patterns in Studies 1 and 2 is evident from 3-dimensional vector plots (Fig. 1). For example, the factor of aggression, indicated in red, consists of the items of hitting, kicking, biting, scratching, and throwing things in both studies. In a 3-dimensional space, vectors for the aggression items are mostly determined by their x-axis value (aligning with the x-axis), which means that they are visually most pronounced in the front and top view images (first and second row), in which the vector space is observed with the z-axis pointing toward the viewer and with the y-axis pointing toward the viewer, respectively. Similarly, factors indicated in green (restlessness) and blue (negativity) also share items between the two studies and predominantly align with axes y and z, respectively. Items that comprise the fourth factor (Combination, purple) are visible to a similar extent from all 3 perspectives, which means that there is no axis (in a 3-dimensional space) with which they align. Finally, like the analysis by Rabinowitz et al.[23], vectors for hiding and hoarding do not follow any of these 4 patterns.

It is also evident that each vector (i.e., each CMAI item) contributes to the overall CMAI construct, as evidenced by similar lengths of vectors aligned with each axis. In other words, the overall vector space is not dominated by any individual factor. This is consistent with the observation by Rabinowitz et al. [23] that the 4 identified factors explain only about half of the variance in Studies 1 and 2, respectively (Table 2). Additional factors would be needed to explain a substantial part of the variance (ideally targeting about 80 % explained). The four vector clusters that can be identified in our plots are coherent semantically with the factors identified by Rabinowitz et al. [23]: items in the aggressiveness cluster correspond to Factor 1 (aggressive behavior), restlessness corresponds with Factor 2 (physically nonaggressive behavior), the negativity cluster corresponds with Factor 3 (verbally agitated behavior), and the combination cluster includes items corresponding to Factors 1, 2 and 3.

**Fig. 2.**
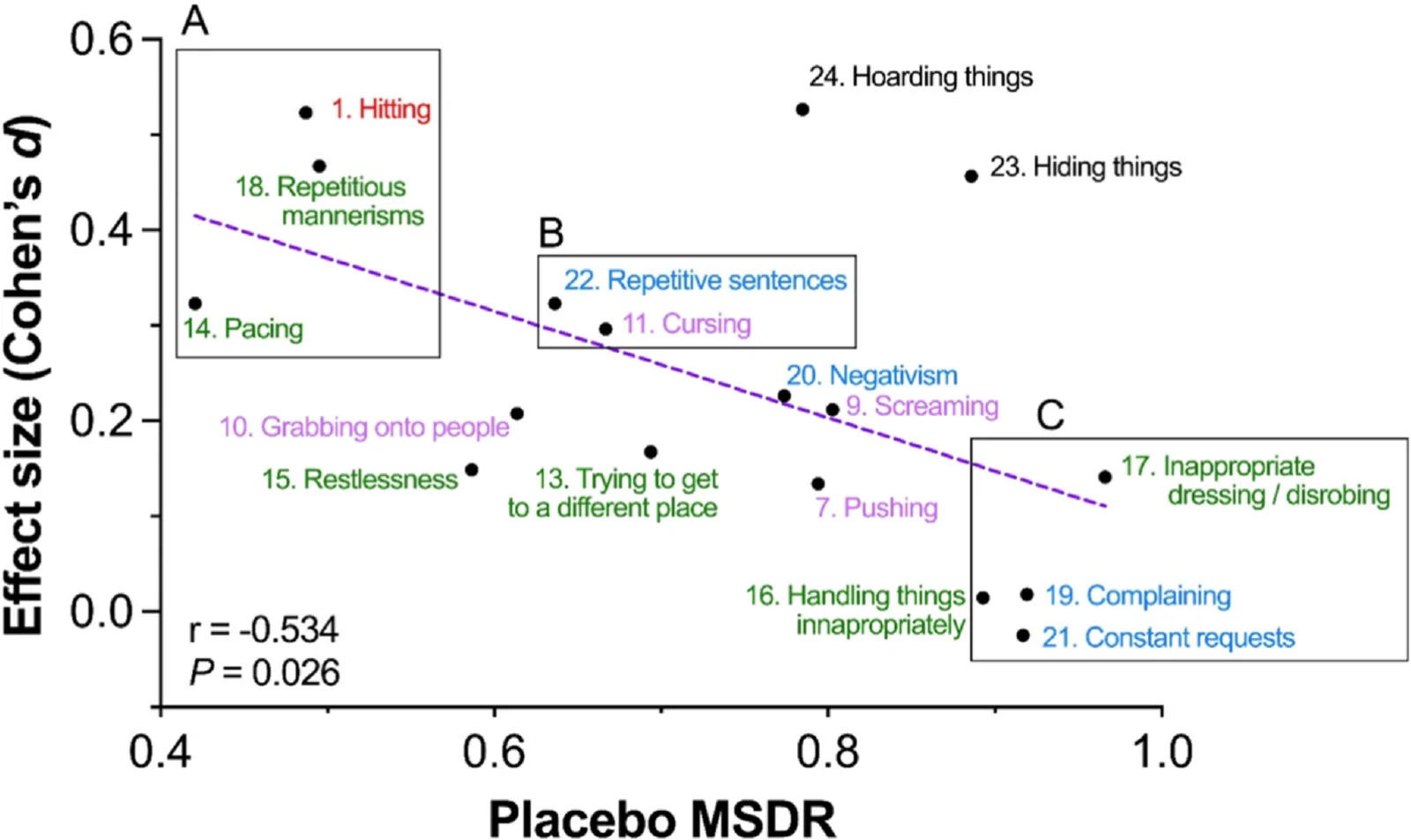
CMAI physical symptoms with clinically meaningful levels at baseline in patients with psychosis of AD: relationship between signal-to-noise ratio in the placebo group and the percentage slowing of decline with risperidone. *For each item, slowing of decline with risperidone was determined as percentage improvement versus placebo, calculated as follows: $\frac{CFB\left(R\right)-CFB\left(P\right)}{CFB\left(P\right)}*100$ Color coding corresponds with factors into which each item loaded, as described in Fig. 1 (red, Aggression; green, Restlessness; blue, Negativity; pink, Combination). Boxes A, B, and C indicate items with greatest to lowest effect sizes, respectively, and also the highest to lowest variation between patients. The items of Hoarding and Hiding, which did not load into any factor, show the largest deviation from this linear relationship. CFB, change from baseline; CMAI, Cohen-Mansfield Agitation Inventory; MSDR, mean-to-standard-deviation ratio; P, placebo; R, risperidone.

In addition, CMAI physical items that vary the most among all patients with psychosis of AD (i.e., those with the smallest mean-to-standard-deviation ratios, as measured in the placebo group) were also the items with the greatest effect sizes following risperidone treatment, and vice versa (correlation coefficient: −0.76; Fig. 2). The three items most responsive to treatment, hitting, repetitive behavioral mannerisms, and aimless pacing or wandering, belong to different factors. This also supports the view that the overall CMAI score is not dominated by any one factor (Fig. 2). The items of hiding and hoarding deviated the most from this linear relationship.

## Discussion

Our findings favor the use of the total CMAI score for monitoring of agitation and aggression in clinical trials and practice involving patients with psychosis of AD.

First, our vector-space analysis is consistent with the distribution of CMAI items across 4 factors, as observed by Rabinowitz et al. and with their estimate that those factors account for only about a half of the data variance in studies they used [23]. Because of such a low variance coverage, it can be argued that, in patient assessment, meaningful information can be lost by using the factors or domain subscores instead of the total CMAI score. Further, mapping of certain CMAI items onto the factors is relatively weak (i.e., they align with 2 or more factors). The discrepancy in responsiveness to treatment between items and the spread of responsive items across factors suggest that some important aspects of agitated or aggressive behavior, such as complaining or inappropriate dressing or disrobing, would be neglected in an assessment based only on factor analysis-derived subscores. In other words, a total CMAI score would reduce the loss of symptom representation that is inherent to the domain-based approach.

Second, we observed that the agitative-aggressive behaviors with the lowest signal-to-noise ratio were most responsive to the effects of risperidone. This stands in contrast with cognitive assessments, in which items with the highest signal-to-noise ratio in the placebo group tend to show the largest treatment in response to disease-modifying therapies [17]. This observation raises interesting analytic possibilities. For example, the effect of risperidone in individuals with dementia is likely only symptomatic, and there may be a discrepancy between items based on their ability to respond to disease-modifying versus symptomatic treatments. (A systematic evaluation of this question, which would require analysis of cognitive and behavioral measures from AD trials of symptomatic and disease-modifying therapies across the disease spectrum, is beyond the scope of the current manuscript.) Alternatively, we speculate that behavioral symptoms may have a different pattern of response to treatment, compared to cognitive symptoms, because of the underlying brain circuitries and their adaptations to neurodegeneration and treatment [18,27]. It is also possible that the CMAI items represent a continuum of behavioral disturbances ranging from mild (e.g., complaining) to moderate (verbal aggression) and severe (throwing things, hitting), and their frequency, as well as signal-to-noise ratio, is likely to depend on disease severity [10,22]. Regardless of the underlying biology, our data indicate that the three CMAI items with the greatest effect sizes (hitting, performing repetitious mannerisms, hoarding things) belong to different factors identified in Studies 1 and 2, which suggests that the total CMAI score captures clinically useful information that a separate factor subscore would miss.

As an illustration, focusing on these three most responsive items may be appropriate in clinical trials of patients with early AD; in such trials, observed worsening (cognitive, functional, or behavioral) is relatively small, and signal maximization is of great importance. In this context, the total CMAI score would be applicable to a much broader range of AD severity.

While we advocate for the use of the total CMAI score, two possible exceptions are the items of hiding things and hoarding behavior, because of their small contribution to the principal component analysis.

In addition to the CMAI scale, a similar analysis of the Neuropsychiatric Inventory – Clinician Rating Scale (NPI-C) or, specifically, Neuropsychiatric Inventory Agitation/Aggression domain (NPI-A-A), would be of interest, especially because newer versions of these scales (CMAI-IPA and NPI-IPA-A-A) have been proposed to incorporate International Psychogeriatric Association (IPA) criteria [8,11].

While our analysis is based on a relatively large number of patients from two studies conducted in several countries (N = 648), there are limitations to be considered when interpreting these findings. First, all patients had severe dementia (MMSE < 10) and severe enough agitation that they were eligible for a pharmacological trial. The finding may not be replicable in a cohort that is less impaired and possible receiving non-pharmacological treatment. Second, the patients came from affluent, predominantly English-speaking societies, and were predominantly White. Evaluating patient groups of different backgrounds, which could manifest as different age, women-to-men ratio, or medication status, would be necessary for generalizing our findings. CMAI studies conducted in societies similar in affluence and the dominance of English language can, and do, produce different factor structures (e.g., [21,23]). Third, data in our analysis stems from clinical trials of risperidone, an atypical antipsychotic. It is feasible that other medications could elicit a different pattern of the CMAI response. Fourth, sensitivity testing, for example, via multidimensional item response theory [25,26,4], principal component analysis using zero-inflated Poisson distributions [14], or multidimensional confirmatory factor analysis [20], which was beyond the scope of our analysis due to reliance on summary data, may yield different results. Fifth, due to data availability, for the analysis of relationship between signal-to-noise ratio versus effect size, we had to focus on the CMAI physical items with a clinically meaningful score at baseline in patients with psychosis of AD. This preselection may have increased signal-to-noise ratio and thereby strengthened the correlation coefficient, compared to an analysis of all the items within the entire patient pool of Studies 1 and 2 [2,9]. Finally, the CMAI items might perform differently in different settings, potentially limiting the generalizability of our findings. For example, Kupeli et al. [21] found that in patients with dementia admitted to general hospitals in London, United Kingdom, the CMAI showed two main types of agitation: aggressive agitation and non-aggressive agitation [21]. Many of the items in the original 29-item CMAI did not appear in the participants in their sample, which resulted in a two-factor, nine-item scale. Finally, there was not biological confirmation of the diagnosis of AD in the studies used, and studies including participants with confirmed diagnoses may have different CMAI scores and profiles.

In conclusion, our findings support the use of the total CMAI score for assessment of agitation and aggression in AD, followed by separate descriptive assessment of each domain, with applicability to a broader range of patients than an assessment based on subsets of items.

## Figures and Tables

**Table 1:** Key Baseline Characteristics of Participants in Studies 1, 2, and 3.

	Study 1[2] N = 309^a^	Study 2[9] N = 344	Study 3[19] N = 625
Women, n (%)	222 (73)	194 (56)	424 (68)
Age, years	82 ± 10^b^	81 (56 –97)^c^	83 ± 8^d^
White	299 (98)	547 (99)	554 (89)
Alzheimer’s dementia, n (%)	180 (59)	229 (67)	456 (73)
Vascular dementia, n (%)	88	90 (26)	97 (16)
Mixed dementia, n (%)	41	25 (7)	72 (12)
MMSE, total score	5.5 ± 8.0^b^	8.4^e^	6.6 ± 6.3^d^

CI, confidence interval; MMSE, Mini Mental State Exam; SD, standard deviation; SEM, standard error of the mean.

a A total of 345 patients were randomized, 337 included at least 1 dose of study drug, and baseline characteristics were calculated for patients from included sites only.

b Mean ± SD (estimated based on mean and SEM values for each group)

c Median (range)

d Mean ± SD

e Mean (original paper cites 95 % Cis for each treatment group, which we did not consider informative enough to provide data spread estimate for the total sample)

**Table 2:** Loading of CMAI items into factors and variance explained by each factor in Australian/New Zealand (N = 304) and Canadian/European data (N = 344), adapted from [23], 25 items included.

	Study 1 (Australia and New Zealand)				Study 2 (Canada and Europe)			
	F1	F2	F3	F4	F1	F2	F3	F4
**CMAI item**								
Hitting (including self)	0.79	−0.03	−0.11	0.05	0.77	−0.02	0.01	0.04
Kicking	0.75	0.03	−0.15	−0.04	0.76	−0.03	−0.04	0.05
Biting	0.75	−0.11	0.07	0.04	0.67	−0.04	−0.11	−0.11
Scratching	0.72	−0.2	0.04	0.08	0.7	0.05	−0.16	−0.11
Throwing things	0.69	−0.09	0.06	0.06	0.58	0.05	−0.04	−0.04
Spitting (include at meals)	0.53	−0.08	−0.09	−0.02	0.56	−0.03	−0.18	−0.18
Pushing	0.51	0.28	−0.1	−0.02	0.65	0.07	0.05	0.05
Tearing things/destroying property	0.47	0.2	−0.05	0.16	0.30	0.27	−0.11	0.34
Screaming	0.45	0.14	0.29	0.13	0.49	−0.30	0.35	−0.05
Grabbing onto people	0.44	0.37	−0.04	−0.21	0.61	0.21	0.09	−0.08
Cursing or verbal aggression	0.4	−0.08	0.4	−0.04	0.42	−0.17	0.31	0.19
Hurting self or others	0.32	0.24	−0.01	−0.25	0.68	0.07	0.06	0.06
Trying to get to a different place	−0.16	0.85	−0.02	−0.03	0.06	0.67	0	−0.08
Pacing, aimless wandering	−0.16	0.75	−0.03	0.15	−0.15	0.72	0.09	−0.02
General restlessness	−0.08	0.73	0.22	−0.09	0	0.61	0.41	−0.04
Handling things inappropriately	0.08	0.63	−0.19	0.17	0.12	0.41	−0.16	0.28
Inappropriate dressing or disrobing	0.20	0.43	0.04	0.38	0.14	0.61	−0.09	0.05
Performing repetitious mannerisms	0.11	0.37	0.24	−0.03	0.17	0.22	0.33	0.04
Complaining	−0.05	−0.07	0.80	0.13	−0.08	−0.11	0.73	0.08
Negativism	0.11	0.12	0.74	0.15	0.05	−0.16	0.63	0.15
Constant unwarranted requests for attention/help	−0.16	0.11	0.73	0.03	0.12	0.16	0.75	−0.14
Repetitive sentences or questions	−0.13	0.16	0.73	−0.05	0.07	0.16	0.68	−0.10
Hiding things	0.04	0.09	0.10	0.86	−0.12	0.02	0	0.84
Hoarding things	0.05	0.13	0.08	0.86	−0.1	−0.04	0.04	0.85
Strange noises (weird laughter/cry)	0.23	0.21	0.19	−0.42	0.26	−0.23	0.12	−0.12
**Variance explained by each factor, %**	**19.4**	**13**	**10.1**	**6.9**	**19.3**	**10.8**	**9.7**	**6.3**

F1: factor 1, Aggressive behavior; F2: factor 2, Physically nonaggressive behavior; F3: factor 3, Verbally agitated behavior; F4: factor 4, Hiding and hoarding.

## References

[1] Brett L, Traynor V, Meedya S, Stapley P. Impressions of using the Cohen-Mansfield Agitation Inventory as an outcome measure: Lessons learnt for future clinical researchers (innovative practice). Dement (Lond) 2020;19:464–71. 10.1177/1471301217695910 29149790

[2] Brodaty H, A randomized placebo-controlled trial of risperidone for the treatment of aggression, agitation, and psychosis of dementia. J Clin Psychiatry 2003;64:134–43. 12633121 10.4088/jcp.v64n0205

[3] Brown BL, Hendrix SB, Hedges DW, Smith TB. Multivariate Analysis for the Biobehavioral and Social Sciences: A Graphical Approach. Wiley,; 2011.

[4] Chalmers RP. mirt: a multidimensional item response theory package for the R environment. J Stat Softw 2012;48:1–29.

[5] Choi SSW, Budhathoki C, Gitlin LN. Co-occurrence and predictors of three commonly occurring behavioral symptoms in dementia: agitation, aggression, and rejection of care. Am J Geriatr Psychiatry 2017;25:459–68. 27914870 10.1016/j.jagp.2016.10.013

[6] Cohen-Mansfield J. Instruction Manual For the Cohen-Mansfield Agitation Inventory. Rockville, MD, USA: The Research Institute of the Hebrew Home of Greater Washington,; 1991.

[7] Cohen-Mansfield J, Marx MS, Rosenthal AS. A description of agitation in a nursing home. J Gerontol 1989;44:M77–84. 2715584 10.1093/geronj/44.3.m77

[8] Cummings J, Agitation in cognitive disorders: International Psychogeriatric Association provisional consensus clinical and research definition. Int Psychogeriatr 2015;27:7–17. 25311499 10.1017/S1041610214001963 PMC4301197

[9] De Deyn PP, A randomized trial of risperidone, placebo, and haloperidol for behavioral symptoms of dementia. Neurology 1999;53:946–55. 10496251 10.1212/wnl.53.5.946

[10] De Mauleon A, Longitudinal Course of Agitation and Aggression in Patients with Alzheimer’s Disease in a Cohort Study: Methods, Baseline and Longitudinal Results of the A3C Study. J Prev Alzheimers Dis 2021;8:199–209. 33569568 10.14283/jpad.2020.66

[11] De Mauleon A, Agitation in Alzheimer’s disease: novel outcome measures reflecting the International Psychogeriatric Association (IPA) agitation criteria. Alzheimers Dement 2021;17:1687–97. 34132461 10.1002/alz.12335 PMC9292260

[12] Finch H. Comparison of the performance of varimax and promax rotations: factor structure recovery for dichotomous items. J Educ Meas 2006;43:39–52.

[13] Halpern R, Seare J, Tong J, Hartry A, Olaoye A, Aigbogun MS. Using electronic health records to estimate the prevalence of agitation in Alzheimer disease/dementia. Int J Geriatr Psychiatry 2019;34:420–31. 30430642 10.1002/gps.5030 PMC7379654

[14] Hellton KH, The truth behind the zeros: a new approach to principal component analysis of the neuropsychiatric inventory. Multivar Behav Res 2021;56:70–85. 10.1080/00273171.2020.1736976 PMC8867488 32329370

[15] Hendrix SB. Measuring clinical progression in MCI and pre-MCI populations: enrichment and optimizing clinical outcomes over time. Alzheimers Res Ther 2012;4:24. 22805433 10.1186/alzrt127 PMC3506938

[16] Hendrix SB, Optimizing measurement of agitation and aggression in dementia. Alzheimer’s Dement 2017;13:264.

[17] Hendrix SB, Nicodemus-Johnson J, Kowallis L, Knowlton N, Hennessey S, Dickson SP. Statistical considerations in the design and analysis of Alzheimer’s disease clinical trials. In: Cummings J, Kinney J, Fillit H, editors. Alzheimer’s Disease Drug Development: Research and Development Ecosystem. Cambridge, UK: Cambridge University Press; 2022. p. 232–48.

[18] Kang W, Wang J, Malvaso A. Inhibitory control in aging: the compensation-related utilization of neural circuits hypothesis. Front Aging Neurosci 2021;13:771885. 35967887 10.3389/fnagi.2021.771885 PMC9371469

[19] Katz IR, Jeste DV, Mintzer JE, Clyde C, Napolitano J, Brecher M. Comparison of risperidone and placebo for psychosis and behavioral disturbances associated with dementia: a randomized, double-blind trial. Risperidone Study Group J Clin Psychiatry 1999;60:107–15. 10084637 10.4088/jcp.v60n0207

[20] Kline RB. Principles and Practice of Structural Equation Modeling. New York, London: The Guilford Press,; 2016.

[21] Kupeli N, Psychometric evaluation of the Cohen-Mansfield Agitation Inventory in an acute general hospital setting. Int J Geriatr Psychiatry 2018;33:e158–65. 28560807 10.1002/gps.4741

[22] McCarty HJ, Longitudinal course of behavioral problems during Alzheimer’s disease: linear versus curvilinear patterns of decline. J Gerontol A Biol Sci Med Sci 2000;55:M200–6. 10811149 10.1093/gerona/55.4.m200

[23] Rabinowitz J, Davidson M, De Deyn PP, Katz I, Brodaty H, Cohen-Mansfield J. Factor analysis of the Cohen-Mansfield Agitation Inventory in three large samples of nursing home patients with dementia and behavioral disturbance. Am J Geriatr Psychiatry 2005;13:991–8. 16286443 10.1176/appi.ajgp.13.11.991

[24] Rabinowitz J, Katz I, De Deyn PP, Greenspan A, Brodaty H. Treating behavioral and psychological symptoms in patients with psychosis of Alzheimer’s disease using risperidone. Int Psychogeriatr 2007;19:227–40. 16879763 10.1017/S1041610206003942

[25] Reckase MD. Multidimensional Item Response Theory. New York: Springer-Verlag,; 2009.

[26] Reise SP, Revicki DA. Handbook of Item Response Theory Modeling: Applications to Typical Performance Assessment. New York: Routledge, Taylor & Francis Group; 2015.

[27] Samson RD, Barnes CA. Impact of aging brain circuits on cognition. Eur J Neurosci 2013;37:1903–15. 23773059 10.1111/ejn.12183 PMC3694726

[28] Sano M, Agitation in cognitive disorders: Progress in the International Psychogeriatric Association consensus clinical and research definition. Int Psychogeriatr 2024;36:238–50. 36880250 10.1017/S1041610222001041 PMC10684256

[29] Schwertner E, Behavioral and psychological symptoms of dementia in different dementia disorders: a large-scale study of 10,000 individuals. J Alzheimers Dis 2022;87:1307–18. 35491774 10.3233/JAD-215198 PMC9198804

[30] Selbaek G, Engedal K, Benth JS, Bergh S. The course of neuropsychiatric symptoms in nursing-home patients with dementia over a 53-month follow-up period. Int Psychogeriatr 2014;26:81–91. 24059840 10.1017/S1041610213001609

[31] Wong B, Wu P, Ismail Z, Watt J, Goodarzi Z. Detecting agitation and aggression in persons living with dementia: a systematic review of diagnostic accuracy. BMC Geriatr 2024;24:559. 38926638 10.1186/s12877-024-05143-6 PMC11210082

